# Obituary

**Published:** 1848-11

**Authors:** 


*Obituary.*—Died, by the accidental discharge of his own gun, on the 13th ult., George Sprague, medical student, son of Alden S. Sprague, M. D., of this city, aged nineteen years.

The death of this promising young man, under such circumstances, occasioned a deep sensation in this community, and called forth heart felt sympathies with the bereaved parents. Possessing excellent talents and acquirements, a fine person, united with habits of diligent application, and moral qualities in every way unexceptionable, the deceased gave every promise of becoming, in after life, all that friend and parent could desire. Arrested, without an instant's premonition, at the threshold of his career, reason and consolation have no alternative but acquiescence in the inscrutable providences of the Divine Author of all human events, who is infinite in His mercy, as well as in His power and wisdom. Funeral solemnities were held at the Pearl St. Church on the 16th ult, on which occasion an appropriate and eloquent discourse was pronounced by the pastor of the church, the Rev. Dr. Lord, which, we are gratified to learn, is to be published.

